# Measles

**Published:** 1883-06

**Authors:** 


### MEASLES.

MEASLES prevails extensively in cities during the winter season, and will usually cure itself, if only protected against adverse influences. The older persons are, the less likely they are to recover perfectly from this ailment, for it very often leaves some life-long malady behind it. The most hopeless forms of consumptive disease are often the result of ill-conducted or badly managed measles. In nine cases out of ten not a particle of any medicine is needed.

Our first advice is always, and under all circumstances, send for a physician of experience at once. Meanwhile keep the patient in a cool, dry and well-aired room, with moderate covering, in a position where there will be no exposure to draughts of air. The thermometer should range at about sixty five degrees, where the bed stands, which should be moderately, hard, of shucks, straw, or curled hair. Gratify the instinct for cold water and lemonade. It is safest to keep the bed for several days after the rash has begun to die away. The diet should be light, of an opening, cooling character.

The main object of this article is to warn persons that the great danger is after the disappearance of the measles. We would advise that for three weeks after the patient is well enough to leave his bed, he should not go out of the house, nor stand or sit for a single minute near an open window or door, nor wash any part of the person in cold water, but to wipe the face with a damp cloth.

For a good part of this time the appetite should not be wholly gratified; the patient should eat slowly of light, nutritious food. In one case, a little child, almost well of measles, got to playing with its hands in cold water; it gradually dwindled away and died.

All exercise should be moderate in order to prevent cooling off too quickly afterward, and to save the danger of exposure to draughts of air, which, by chilling the surface, cause *chronic diarrhœa*, if it falls on the bowels; *deafness for life*, if it falls on the ear; or *incurable consumption*, if it falls on the lungs.

In view of these hazards, it is important to be able to recognize the disease on its first appearance. The disease with which it is most likely to be confounded is scarlet fever.

Measles usually commence with a feeling of lassitude, chilliness and aching of the limbs, followed by frequent pulse, heat and dryness of the skin, loss of appetite and furred tongue. There is often irritation of eyes and nostrils with sneezing, hoarseness and cough.

The eruption generally appears about the fourth day, though it may be a longer or shorter time.

The rash occurs at first in small, red, distinct spots, like flea bites, which *disappear* under pressure. These show themselves first in the face and neck; then upon the trunk, and finally upon the limbs.

Scarlet fever is characterized by inflammation of the throat and by the eruption appearing first on the neck instead of the face, as with measles, and by the fact that the eruption is more diffused, and does not disappear on pressure as in measles.

Although there is more or less fever in measles, there is an almost entire absence of inflammatory symptoms that characterize scarlet fever; still it is doubtful if the accidents that follow the disease are more frequent or more deplorable than those that follow, even the milder forms of measles, when neglected and improperly treated.

---

THE best three medicines in the world are warmth, abstinence, and repose.

STOMACH ACHE, from indigestion, may generally be relieved by lying on the back and rubbing the abdomen with the hands, pressing and rubbing downward.

IN walking or other exercise, learn to keep the mouth firmly closed, and to breathe entirely through the nose. You can walk as far again with less fatigue and without getting out of breath, than when you breathe through the mouth. Try it.

